# Bibliographical Notices

**Published:** 1849-02

**Authors:** 


### BIBLIOGRAPHICAL NOTICES.

*A Dispensatory, or Commentary on the Pharmacopœias of Great Britain and the United States, comprising the Natural History, Description, Chemistry, Pharmacy, Actions, Uses, and Doses of the Articles of the Materia Medica.* By ROBERT CHRISTISON, M. D., etc, Second Ed. Revised and improved, &c., with 213 illustrations. By R. E. GRIFFITH, M. D., etc. Philadelphia; Lea & Blanchard: 1848. pp. 1008.

The science of Therapeutics has not advanced as rapidly, of late, as that of Pathology and Physical Diagnosis, and we therefore welcome any attempt at its advancement, especially from such a source as the work, whose title we have given. We fully agree in opinion with the editor of the Charleston Medical Journal, that a philosophical treatise on the elements or principles of Therapeutics, is still a desideratum in our language. We have nothing approaching such a work as is demanded by the existing state of the other branches of medicine;—nothing which presents a clear, consistent and correct account of the modus operandi, the physiological effects, and the therapeutical applications of remedial agents. We trust the time is not remote, when this want will be satisfactorily met.

We are pleased with Dr. Christison's work, because it is in advance of most that has been written on the subject. It is not altogether a re-hash of what others have said and written, but contains much that is the result of the author's personal observation at the bed-side, and in the treatment of the sick. The student and the practitioner, are not so much concerned in the botanical, chemical, pharmaceutical and commercial history of drugs, as with their physiological action, and their application to the treatment of disease. The former are not without a certain degree of interest to the medical man, but an acquaintance with them furnishes him no aid in curing the sick; and at this day, when the duration of life seems half adequate to learn and do what seems indispensible to know and to perform, it is the part of wisdom to occupy the mind with what we can turn to some practical use.

The work of Dr. Christison is constructed upon the same plan with that of Wood & Bache, and does not differ materially from it, in its mode of treating the different subjects. The remarks upon the different articles and preparations of the materia medica, are full, copious, and sufficiently practical; had the latter been extended even at the expense of the former, the work would not, in our estimation, have suffered in value. As it is, it will be found a very useful addition to the library of every practitioner.

The editor has performed his task with his well known, eminent ability, having added all the processes of the United States Pharmacopœia, and a description of such articles as are recognized in that work, together with many useful tables, and numerous illustrations of medicinal plants, apparatus, &c. The work is for sale at Derby's Bookstore, where all standard medical works may also be had. C. A. L.

---

*Notes on Medical Matters, and Medical Men, in London and Paris.* By David W. Yandell, M. D. Louisville, Ky. Prentice & Weissinger. 1848. 8vo. pp. 309.

The author of the above is a son of Prof. Yandell, of the Louisville Medical College, who has recently returned from Paris, having resided abroad for two years to complete his medical education. The work consists of a series of letters published, during his residence at Paris, in the Western Journal of Medicine and Surgery. The volume is made up of extra sheets of the letters, set apart for that purpose, as the successive numbers were passing through the press. It is not intended for sale, but for distribution among his friends.

We are indebted to the author for his kindness in transmitting a copy to us. Our readers will recall several valuable extracts from these letters, copied, from time to time, into our pages. They evince a spirit of inquiry, an aptness for observation, and a discriminating judgment, which, with zeal and application, cannot fail to acheive success. Dr. Yandell is quite young. Let him remember that youthful laurels are chiefly desirable and valuable as incentives to aspirations, and industry, which recognise, as a limit to farther exertions, no *ultimatum* of success. It is with every department of knowledge, as Cicero declared it to be with the orator—he who would superlatively excel, must ever aim at what he can never reach—*aliquid immensum, infinitumque.*

---

*Lectures on Venereal, and other Diseases arising from Sexual Intercourse. Delivered in the summer of* 1847, *at the Hopital du Midi., Paris.* By M. Ricord. Reported and translated by Victor De Maric, M. D., M. R. C. S. E. Philadelphia. Ed. Barrington & Geo. D. Hazwell. 1849. 12 mo. pp. 298.

This volume consists of lectures, first published in the London Lancet for 1847–8, which are said to have been reported with fidelity. The latter is all that the reader will require to be assured of, the name of Ricord being a sufficient guaranty for the rest.

---

*Principles of Medicine: Comprising General Pathology and Therapeutics, and a brief general view of Etiology, Nosology, Semeiology, Diagnosis, Prognosis, and Hygienics.* By Charles J. B. Williams, M. D., F. R. S., etc. Edited, with additions, by Meredith Clymer, M. D., etc. Third American from the second and enlarged London Edition. Philadelphia: Lea & Blanchard. 1848. 8vo. pp. 440.

The above is the title of a new edition of a work well known to the Profession, and, deservedly held in high estimation. For sale by Derby & Co.

